# Accelerated ovarian failure results in brain alterations related to Alzheimer's disease that are not recovered by high‐intensity interval training in mice

**DOI:** 10.1002/alz.14463

**Published:** 2024-12-23

**Authors:** Ahmad Mohammad, Michael S. Finch, Sarah Rouhana, Parastoo Mashouri, Ciara Barry, Emma F. Hubbard, Newman Sze, Shawn M. Beaudette, W. Glen Pyle, Geoffrey A. Power, Rebecca E. K. MacPherson

**Affiliations:** ^1^ Department of Health Sciences Brock University St. Catharines Ontario Canada; ^2^ IMPART Team Canada Dalhousie Medicine Dalhousie University Saint John New Brunswick Canada; ^3^ Department of Human Health and Nutritional Sciences College of Biological Sciences University of Guelph Guelph Ontario Canada; ^4^ Department of Kinesiology Brock University St. Catharines Ontario Canada; ^5^ Centre for Neuroscience Brock University St. Catharines Ontario Canada; ^6^ Centre for Bone and Muscle Health Brock University St. Catharines Ontario Canada; ^7^ Women's Health Research Institute B.C. Women's Hospital + Health Centre Vancouver British Columbia Canada

**Keywords:** brain, estrogen, exercise, ovarian failure

## Abstract

**INTRODUCTION:**

The menopausal decline in ovarian estrogen production is thought to increase the risk of Alzheimer's disease; however, this link requires further investigation. The chronological development of this connection is not well defined because of the lack of animal models that recapitulate the time course of menopause. This study characterized the cognitive and neuronal effects of the 4‐vinylcyclohexene diepoxide (VCD) model of ovarian failure in female mice and assessed whether high‐intensity interval training (HIIT) would attenuate impairments.

**METHODS:**

Female mice were injected with VCD for 15 days. Novel object recognition tests (NORT) were conducted during (perimenopause) and after (menopause) ovarian failure (*n* = 7). HIIT was initiated in menopause and mice underwent NORT testing after 2 and 8 weeks of HIIT (*n* = 5).

**RESULTS:**

VCD mice had a lower discrimination index, and lower SNAP25 and NeuN expression in perimenopause. HIIT did not recover memory in VCD mice.

**DISCUSSION:**

Neuronal changes arise early in the perimenopausal transition and HIIT did not improve recognition memory when initiated in menopause.

**Highlights:**

- The menopausal decline in ovarian estrogen production increases the risk of Alzheimer's disease (AD).
- The chronological development of this connection is not well defined because of the lack of animal models that recapitulate the time course of menopause.
- 4‐vinylcyclohexene diepoxide (VCD)–induced ovarian failure provides a model that simulates the average human experience in the transition from perimenopause to menopause.
- We demonstrate that cognitive and biochemical effects related to AD pathology are present from the earliest available timepoint in perimenopause in VCD mice.
- This work highlights the importance of examining the time course in the progression to menopause and the use of VCD as a model to investigate changes in the brain.

## 1 BACKGROUND

Alzheimer's disease (AD) is a debilitating neurodegenerative disease that disproportionately affects females, who represent ≈ 70% of cases.^1^, ^2^ AD has a long prodromal phase that precedes pathological symptoms and often coincides with the onset of menopause.^3^, ^4^ The reduction in ovarian estrogen production that occurs with the menopausal transition is thought to play a key role in the difference in disease prevalence between females and males.^5^ Studies have examined AD progression in late menopausal stages but there is a lack of research examining the memory and brain changes present throughout the transition, specifically those that might arise early in the perimenopausal transition.

To investigate brain changes associated with menopause, animal studies typically use an ovariectomy (OVX) model, in which ovaries are surgically removed to mimic estrogen depletion in menopause. A role for estrogen loss in the pathogenesis of AD has been advanced with OVX studies in which bilateral resection is associated with reductions in cognitive performance.^4^ Studies using the OVX model demonstrate higher amyloid beta (Aβ) peptide production and accumulation, which are hallmarks of AD,^4^, ^6^, ^7^ along with higher β‐secretase (BACE1) activity, the rate‐limiting enzyme in Aβ production.^4^ OVX also results in lower neuronal and synaptic marker content,^4^, ^6^, ^7^ alterations that are commonly observed in AD. AD is associated with lower NeuN content, a marker of mature neurons,^8^, ^9^ indicating that AD causes gradual loss of mature neurons. At the synapse, there are changes in markers related to neurotransmitter release (SNAP25) and proteins involved in scaffolding to maintain synaptic integrity (PSD95).^3^, ^10^ While the OVX model contributes to our knowledge of the connection between ovarian hormone loss and AD pathology in females, it does not mimic the hormonal alterations or the temporal profile of the human menopausal experience.

Most females experience a lowering of ovarian estrogen production that starts between the ages of 45 and 55, leveling off at a significantly reduced level around the ages of 50 to 60.^11^, ^12^ The transitionary phase prior to menopause is known as perimenopause, while the reduced plateau in estrogen is menopause.^13^, ^14^, ^15^ OVX can recapitulate the estrogen‐deficient state of menopause, but it also yields a reduction in all ovarian hormones and the sudden loss of ovarian function does not reflect the gradual, decade‐long perimenopausal phase experienced by most females. A rodent model that better simulates the average human experience is the accelerated ovarian failure (AOF) model.^7^ This model involves the injection of 4‐vinylcyclohexene diepoxide (VCD) to cause gradual ovarian failure and estrogen depletion in rodents, while having no non‐ovarian toxic effects.^16^, ^17^, ^18^ In the AOF model, VCD is injected over 15 days, and early perimenopause is evident ≈ 60 days post‐VCD when estrus cycles are prolonged demonstrating the beginning of this transitionary stage. At 120 days post‐VCD, follicular depletion is complete, and animals are acyclical, marking the onset of the post‐menopausal period. The AOF model has been used to examine changes in muscle health^19^ and heart disease progression;^16^, ^17^, ^20^ however, we currently lack understanding surrounding the changing hormonal milieu through the perimenopausal transition on markers of neurodegeneration.

Given the long timeline in the development of AD, prevention is essential to reduce the incidence of the disease. Exercise can slow AD progression and improve cognitive function.^5^, ^9^, ^21^, ^22^ A common barrier to exercise is a lack of time, and high‐intensity interval training (HIIT), comprised of short high‐intensity exercise interspersed with rest intervals, is an appealing option because of its time efficiency and low cost, which enhances accessibility.^23^ One hypothesis behind the mechanisms by which exercise blunts AD progression is that physical activity increases the non‐pathological degradation of amyloid precursor protein (APP) through a disintegrin and metalloprotease domain 10 (ADAM10) activity while lowering BACE1 activity.^4^, ^5^, ^24^, ^25^, ^26^ This implies that exercise can influence the processing of APP to favor the non‐amyloidogenic cascade, lowering Aβ production.^4^, ^27^, ^28^, ^29^

**RESEARCH IN CONTEXT**

1. **Systematic review**: Alzheimer's disease disproportionately affects post‐menopausal females. This is thought to occur because of the drastic hormonal shift females experience during the menopausal transition.
2. **Interpretation**: Our findings contribute to the neuronal characterization of the 4‐vinylcyclohexene diepoxide menopausal model and establish a time course for neurological changes that develop prior to menopause.
3. **Future directions**: Further analysis of the perimenopausal transition is required to determine the finite timing of the neuronal effects and if exercise interventions implemented prior to the onset of menopause are more effective at reducing post‐menopausal changes.

The first purpose of this study was to characterize the effects of the VCD‐induced AOF model on memory and APP processing throughout the progression from perimenopause to late‐stage menopause. The second purpose was to determine whether a HIIT protocol could attenuate AD‐related changes in VCD‐induced menopause.

## 2 METHODS

### 2.1 Animals and design

All procedures were approved and in accordance with the guidelines set by the Animal Care and Use Committee of the University of Guelph (AUP #4277 for WGP and AUP # 4714 for GAP) and the Canadian Council on Animal Care. Sexually mature female CD‐1 mice were acquired from Charles River Laboratories at 10 to 12 weeks of age and weighing 22 to 25 g. Mice were housed at 24°C with a 12 hour light/12 hour dark cycle and given ad libitum access to food and water. Mice acclimatized to housing conditions for 1 week, after which they were assigned to control or VCD groups. Mice in the VCD groups received intraperitoneal injections with 160 mg/kg body mass of VCD (diluted with sesame oil to a 0.0587:1 ratio, MilliporeSigma) daily for 15 days (sample size was 7–8 per group). This injection protocol leads to ovarian follicular failure over 120 days, after which mice are in an ovarian failure state that mimics human menopause.^17^, ^18^, ^19^ Control groups were injected with vehicle (sesame oil) to simulate the stress of injection. The VCD model of ovarian failure has a well‐characterized hormone profile that closely mimics human menopause, when ≈ 120 days after VCD treatment, plasma estradiol levels become very low in treated mice.^18^ This model has been previously used to examine temporal alterations in the development of cardiovascular disease risk and changes to muscle contractile function associated with menopause.^17^, ^19^, ^20^, ^30^, ^31^ Based on vaginal cytology mice are acyclical by day 120 post‐VCD treatment. Therefore, vaginal cytology was used to confirm mice were in a constant diestrus phase in this study.

Control and VCD mice were characterized via recognition memory and biochemical analysis at varying timepoints in perimenopause and menopause. The perimenopause timepoints, when the follicles are being actively depleted, were at 60 and 120 days post‐injection to represent the middle and end of the transitional phase. The menopause timepoints were 134 and 176 days after injection, with these timepoints representing an early and late menopause period, respectively.^17^ Days are counted starting with the first day of injection.

The second aim of this work was to determine whether an 8‐week HIIT protocol could reverse the neurological effects of VCD‐induced menopause when initiated at the onset of menopause (day 120). For this aim, mice were assigned to either VCD or VCD+HIIT (*n* = 5/group). As described previously, prior to training, mice underwent a 2‐week treadmill familiarization protocol (EXER 3/6 animal treadmill, Columbus Instruments).^19^ In the first week, mice ran for 5 minutes on three separate days, beginning at 5 meters/minute (m/minute) and progressing to a 10 m/minute finish. In the second week mice underwent the same familiarization with a 25° incline. The HIIT protocol started at 120 days post‐VCD injection. The HIIT protocol consisted of 8 weeks of 10 minute sessions at 25° incline, 3 days per week. Each training session consisted of a 3 minute warm‐up at a base speed that was easily maintained by all mice (5 m/minute in weeks 1–2, 6 m/minute in weeks 3–4, 8 m/minute in weeks 5–6, 11 m/minute in weeks 7–8), followed by three 1 minute intervals at a sprint speed with 1 minute of recovery at base speed between sprints (7 m/minute in weeks 1–2, 10 m/minute in week 3–4, 13 m/minute in week 5–6, 16 m/minute in week 7–8), and a final fastest 1 minute dash interval (10 m/minute in weeks 1–2, 15 m/minute in weeks 3–4, 18 m/minute in week 5–6, 21 m/minute in week 7–8).^19^ Intensity was increased every 2 weeks and speeds were adjusted accordingly. This ultimately resulted in mice getting 2 or 8 weeks of HIIT depending on at which timepoint the samples were collected.

### 2.2 Novel object recognition testing

A novel object recognition test (NORT) was performed at each timepoint (60, 120, 134, and 176 days post‐injection) for characterization (Aim 1) and at days 134 and 176 for HIIT mice (Aim 2). The NORT was performed in four individual steps, which involved two habituation phases, one familiarization phase, and one testing period. For all the required steps, the same open arena was used, measuring 40 × 40 × 40 cm. The two habituation phases were run 24 hours apart and involved placing the mice into the same corner of the empty arena and allowing them to explore freely for 10 minutes. Twenty‐four hours later, the mice were put through the second habituation period, after which they were put through the familiarization process. Familiarization involved placing two identical objects in the arena with one in the top right and the other in the top left. The objects were placed 5 cm away from the walls and the mice were allowed to explore the arena for 10 minutes. The familiarization was recorded, and the arenas were sanitized with a virox solution between trials. The mice were then returned to their home cages for a span of 1 hour. During this time, one of the objects within the arenas was replaced with one that would be novel to the mice; that is, they had not been exposed to it previously. Once the hour was complete, the testing stage began, and the mice were allowed to freely explore the arena, now containing one familiar and one novel object, for a span of 10 minutes, while being video recorded. The arenas were cleaned, and the testing was repeated until all the mice had been tested after being familiarized. They were given 72 hours to rest before the entire process was repeated with a gap period of 4 hours instead of 1. During the second NORT, both the familiar and novel objects were changed entirely to ensure that the mice were familiarized with an entirely new object and had a unique novel object as well.

All videos were recorded using a ceiling‐mounted GoPro (1920 × 1080 pixels; 30 fps), with animal tracking performed using DeepLabCut (DLC version 2.2). Specifically, a custom DLC model was trained using user inputs to resolve the locations of four anatomical landmarks (nose, tail base, left ear, and right ear). The DLC model (ResNet 50 architecture) was trained using 20 diverse frames (*k*‐means clustering) from 20 input videos for a total of 200,000 epochs, resulting in a mean testing error of 3.74 pixels across all tracked positional landmarks. Tracked XY positional data from DLC were further analyzed using custom MATLAB (v2022a, The MathWorks Inc.) and to generate discrete performance parameters. Specifically, all XY coordinate data were interpolated using a piecewise cubic Hermite interpolating polynomial to remove periods of low confidence (< 0.9) tracking. Subsequently, anatomical key points were reduced to a weighted spatial mean to represent the center of the head for each mouse. Next, object interaction events were defined when the head marker crossed circular object thresholds with threshold boundaries defined using the maximum length of each object. With interaction periods defined, trials were scored based on the amount of time each mouse spent interacting with each target object, as well as the time spent without interacting with any objects. These time periods were used in the calculation of a discrimination index (%) by calculating the ratio between the time spent investigating the novel object to the total amount of time they spent investigating both objects. A higher value for the ratio indicates that the mice were able to actively remember the familiar object more than the novel one and thus spent more time with the novel object.^4^

### 2.3 Tissue collection

Mice were anesthetized and euthanized by cardiac extraction. Brains were quickly removed, and samples from both the right and left hippocampus (HIPP) and prefrontal cortex (PFC) were isolated and snap frozen in liquid nitrogen. These two brain regions are the regions of the brain first and most significantly affected by AD progression.^32^, ^33^ To confirm the loss of ovarian hormones, each uterus was weighed, and mass was recorded at 120, 134, and 176 days. All isolated tissue was stored in a −80 ° C freezer until further analysis. 

### 2.4 Western blotting 

Both isolated brain regions (HIPP and PFC) were weighed and homogenized in 1 mg:20 µL volumes of NP40 Cell Lysis Buffer (Cat. No. FNN0021, ThermoFisher). Ten mL of NP40 cell lysis buffer was aliquoted before having 50 µL of a protease inhibitor (Cat. No. P2714, Sigma‐Aldrich) and 34 µL of phenylmethylsufonyl fluoride added (Cat. No. P7626, Sigma‐Aldrich). Samples were centrifuged at 4 ° C for 15 minutes at 10,000 g before the supernatant was collected. Protein concentrations were determined using a bicinchoninic acid (BCA) assay. Western blot samples were prepared using 2× Laemmli buffer and a final protein concentration of 1 µg/µL. Fifteen µg of prepared sample was loaded into each well on a 12.5% sodium dodecyl sulfate polyacrylamide gel electrophoresis gel and proteins were electrophoretically separated at 120 V for 90 minutes at room temperature. Proteins were wet transferred from the gel to 0.45 nitrocellulose membranes (Cat. No. 10600002, Cytvia) at 100 V for 60 minutes on ice. Membranes were blocked using 5% non‐fat dry milk‐TBST (Tris‐buffered saline/0.1% Tween 20) for 60 minutes at room temperature. Membranes were then incubated at 4 ° C with slight agitation overnight in a solution containing the appropriate primary antibody diluted 1:1000 in 5% BSA (bovine serum albumin)‐TBST. Primary antibodies were used to determine protein content of BACE1 (Cat. No. 5606S, Cell Signaling), ADAM10 (Cat. No. ab1997, Abcam), APP (Cat. No. 825001, Biolegend), sAPPα (Cat. No. 11088, IBL), sAPPβ (Cat. No. 813401, BioLegend), NeuN (Cat. No. 24307, Cell Signaling), PSD95 (Cat. No. 3450, Cell Signaling), and SNAP25 (Cat. No. 5308, Cell Signaling). The next day, membranes were rinsed three times for a total of 15 minutes in TBST after which they were incubated in the appropriate secondary antibody diluted to 1:2000 in 1% non‐fat dry milk‐TBST for 60 minutes. Membranes were again washed in TBST three times for a total of 15 minutes before proteins were visualized using Western Lightning Plus‐ECL (Cat. No. NEL104001EA, PerkinElmer) in a Bio‐Rad ChemiDoc Imaging System running Image Lab Touch Software. After images were collected, membranes were placed in a ponceau stain (Cat. No. PON002, BIOSHOP) for 10 minutes before being rinsed in distilled water and laid out to dry for imaging. The bands of protein were quantified and analyzed using AlphaView followed by a quantification of the ponceau to ensure equal loading across the membrane (< 10% variability). When analyzing all proteins, the content was made relative to their appropriate ponceau loading control and all phosphorylated proteins were made relative to the total protein content.

### 2.5 BACE activity assay

Homogenates were made at a concentration of 0.5 µg/µL using an extraction buffer. The assays were then loaded and run according to the instructions provided by the manufacturer (Cat. No. ab65357, Abcam) and as previously described.^4^, ^33^, ^34^ Fifty µL of prepared PFC and HIPP samples were loaded into a black 96‐well plate. While the plate was being loaded, the reaction buffer and substrates were preincubated at 37 ° C. Fifty µL of 2× reaction buffer was added to each well followed by 2 µL of BACE substrate. The plate was wrapped in aluminum foil and placed in the dark at 37 ° C for 60 minutes, after which the plate was read using a Spectra Max M2 plate reader at wavelengths of 335 and 496 nm. 

### 2.6 ADAM10 activity assay

ADAM10 activity was detected using the SensoLyte ADAM10 Fluorometric Activity Assay Kit (Cat. No. AS‐72226, SensoLyte) according to the manufacturer's instructions and as previously described.^33^ Fifty µL of homogenized HIPP or PFC at 0.5 µg/µL of total protein was loaded into a black 96‐well plate. Next, 50 µL of diluted ADAM10 substrate was added to each well, and reagents were agitated for 30 seconds by hand. The plate was covered in aluminum foil and placed in the incubator at 37 ° C for 60 minutes in the dark. After incubation, a Spectra Max M2 plate reader was used to read fluorescence at wavelengths of 490 and 520 nm.

### 2.7 Statistical analysis

Graphpad Prism 9 (Version 9) was used for all statistical analysis. All western blots were assessed using a two‐way analysis of variance (ANOVA) with the factors of timepoint (60, 120, 134, and 176 days) and intervention (VCD, HIIT). Differences in all cognitive testing were assessed using a two‐ (single timepoint included and treatment) or three‐way ANOVA (multiple timepoints included, treatment, and inter‐trial interval). Any significant interactions were followed up with a Tukey post hoc analysis. All values that deviated by more than two standard deviations from the mean were considered outliers and removed from the analysis. All significant data were reported as *P* ≤ 0.05. Data in figures are presented as mean values ± standard error of the mean.

## 3 RESULTS

### 3.1 Characterization of the VCD model on AD markers

**FIGURE 1:**
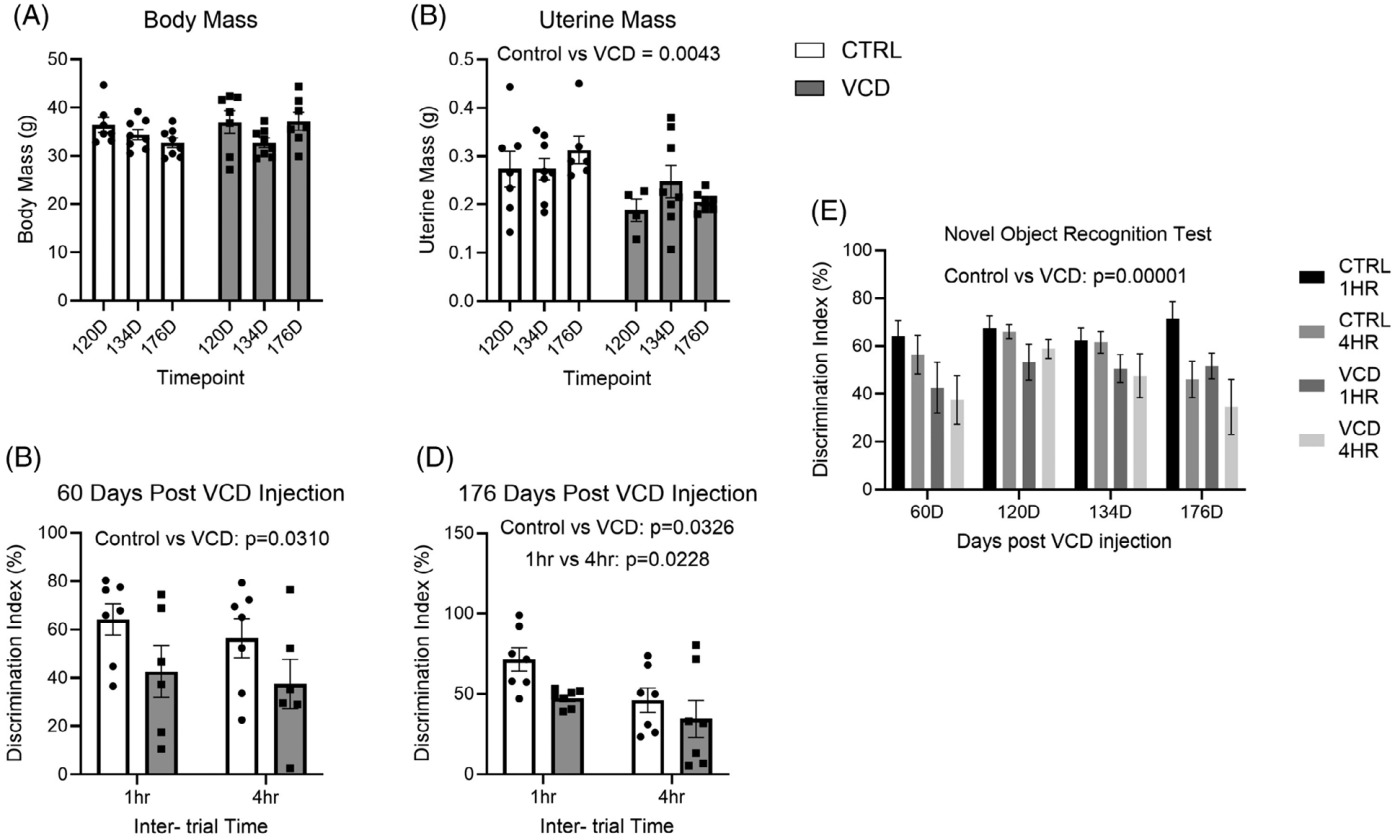
Physical characterization of model alongside cognitive testing. A, Body mass (g). B, Uterine mass (g). C, Sixty day cognitive testing results (discrimination index %). D, One hundred twenty day cognitive testing results (discrimination index %). E, Novel object recognition testing results 60, 120, 134, and 176 days post‐injection using a 1 and 4 hour time interval (discrimination index %). All data were analyzed using a two‐way ANOVA; however, (E) was analyzed using a three‐way ANOVA. *n* = 7 per condition. ANOVA, analysis of variance; CTRL, control; VCD, 4‐vinylcyclohexene diepoxide–treated mice; 60D, 60 days post completion of injection; 120D, 120 days post completion of injection; 134D, 134 days post completion of injection; 176D, 176 days post completion of injection

No differences in body mass were observed between groups at 120, 134, and 176 days post‐VCD injection (Figure 1A). There was a main effect of treatment, such that uterine mass was lower in VCD‐treated mice (CTRL 478.8 ± 115.7 mg vs. VCD 205.7 ± 7.8 mg, *P* = 0.0043; Figure 1B). Decreases in uterine mass is a gold standard method of determining the efficacy of a menopausal model.^4^, ^33^, ^35^ Recognition memory was evaluated via NORT.^35^ We found a main effect for treatment at 60 and 176 days, when VCD mice had a lower % discrimination index (60 days: 1 hour: CTRL 64.2 ± 17.1 seconds vs. VCD 42.6 ± 26.1 seconds; 4 hours: CTRL 56.4 ± 21.2 seconds vs. VCD 37.6 ± 24.8; *P* = 0.0310, Figure 1C; and 176 days: 1 hour: CTRL 71.5 ± 19.0 seconds vs. VCD 47.4 ± 5.5 seconds; 4 hours: CTRL 46.0 ± 20 seconds vs. VCD 34.5 ± 30.5 seconds, *P* = 0.0326, Figure 1D). A main effect of inter‐trial interval time was also observed at the 176‐day timepoint (*P* = 0.0228; Figure 1D). Finally, when comparing all interventions at all timepoints, a three‐way ANOVA demonstrated a main effect for treatment where the VCD‐injected mice have lower discrimination index % across all timepoints (*P* = 0.00001; Figure 1E). The assessment of the 60‐ and 176‐day trials were essential to determine the effects throughout the progression to ovarian failure. Deficits in recognition memory were observed in the VCD mice at each timepoint measured.

### 3.2 APP processing and signalling

**FIGURE 2:**
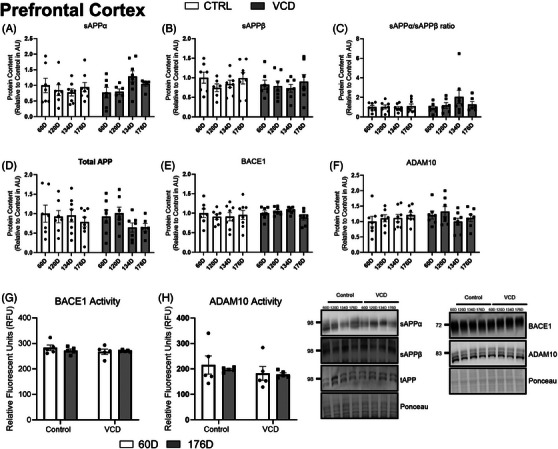
Content of PFC APP markers characterization of the model at different timepoints of VCD progression compared to control. A, sAPPα content (AU). B, sAPPβ content (AU). C, sAPPα/sAPPβ content ratio (AU). D, total APP content (AU). E, BACE1 content (AU). F, ADAM10 content (AU). G, BACE1 activity in relative fluorescence units (RFU). H, ADAM10 activity (RFU). All blots are accompanied by representative blots. All data were analyzed using a two‐way ANOVA. *n* = 7 per condition for western blots, *n* = 5 per condition for activity assays. ADAM10, a disintegrin and metalloprotease domain 10; ANOVA, analysis of variance; APP, amyloid precursor protein; AU, arbitrary scale; BACE1, β‐secretase; CTRL, control; PFC, prefrontal cortex; RFU, relative fluorescence unit; VCD, 4‐vinylcyclohexene diepoxide; 60D, 60 days post completion of injection; 120D, 120 days post completion of injection; 134D, 134 days post completion of injection; 176D, 176 days post completion of injection

To examine amyloidogenic and non‐amyloidogenic processing of APP, sAPPβ and BACE1, as well as sAPPα and ADAM10, were analyzed. In the PFC, there were no differences present between groups when assessing total APP, sAPPα, sAPPβ, sAPPα/sAPPβ ratio, BACE1, or ADAM10 expression (*P* > 0.05). In addition to measuring BACE1 and ADAM10 protein content we also examined their enzymatic activities. BACE1 is the rate‐limiting enzyme in the production of Aβ while ADAM10 is the rate‐limiting enzyme in the non‐amyloidogenic pathway. There were also no differences present in BACE1 or ADAM10 activity (*P* > 0.05; Figures 2A–H).

**FIGURE 3:**
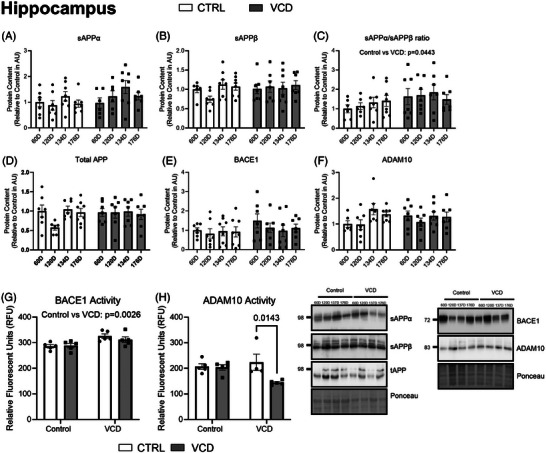
Content of HIPP APP markers in the model at different timepoints of VCD progression compared to control. A, sAPPα content (AU). B, sAPPβ content (AU). C, sAPPα/sAPPβ content ratio (AU). D, total APP content (AU). E, BACE1 content (AU). F, ADAM10 content (AU). (G, BACE1 activity (RFU). H, ADAM10 activity (RFU). All blots are accompanied by representative blots. All data were analyzed using a two‐way ANOVA. *n* = 7 per condition for western blots, *n* = 5 per condition for activity assays. ADAM10, a disintegrin and metalloprotease domain 10; ANOVA, analysis of variance; APP, amyloid precursor protein; AU, arbitrary scale; BACE1, β‐secretase; CTRL, control; HIPP, hippocampus; RFU, relative fluorescence unit; VCD, 4‐vinylcyclohexene diepoxide; 60D, 60 days post completion of injection; 120D, 120 days post completion of injection; 134D, 134 days post completion of injection; 176D, 176 days post completion of injection

In the HIPP, protein content was not different between groups when looking at total APP, sAPPα, sAPPβ, BACE1, or ADAM10 (*P* > 0.05; Figures 3A,B,D–F). There was a main effect of treatment for the ratio of sAPPα to sAPPβ where the VCD groups had a higher ratio (*P* = 0.0443; Figure 3C). BACE1 enzymatic activity was found to be higher in the VCD group (*P* = 0.0026, main effect; 60D: CTRL 286.2 ± 14.0 vs. VCD 326.0 ± 19.5; 176D: CTRL: 288.7 ± 18.9 vs. VCD 311.8 ± 24.9; Figure 3G). For ADAM10 enzyme activity, there was a significant interaction present (*P* = 0.0268) and post hoc analysis demonstrated that there was lower activity in the 176 day (8 week) VCD group compared to the 60D VCD group (VCD 176D 144.1 ± 8.4 vs. VCD 60D 223.9 ± 63.7, *P* = 0.0180; Figure 3H).

### 3.3 Neuronal and synaptic markers

**FIGURE 4:**
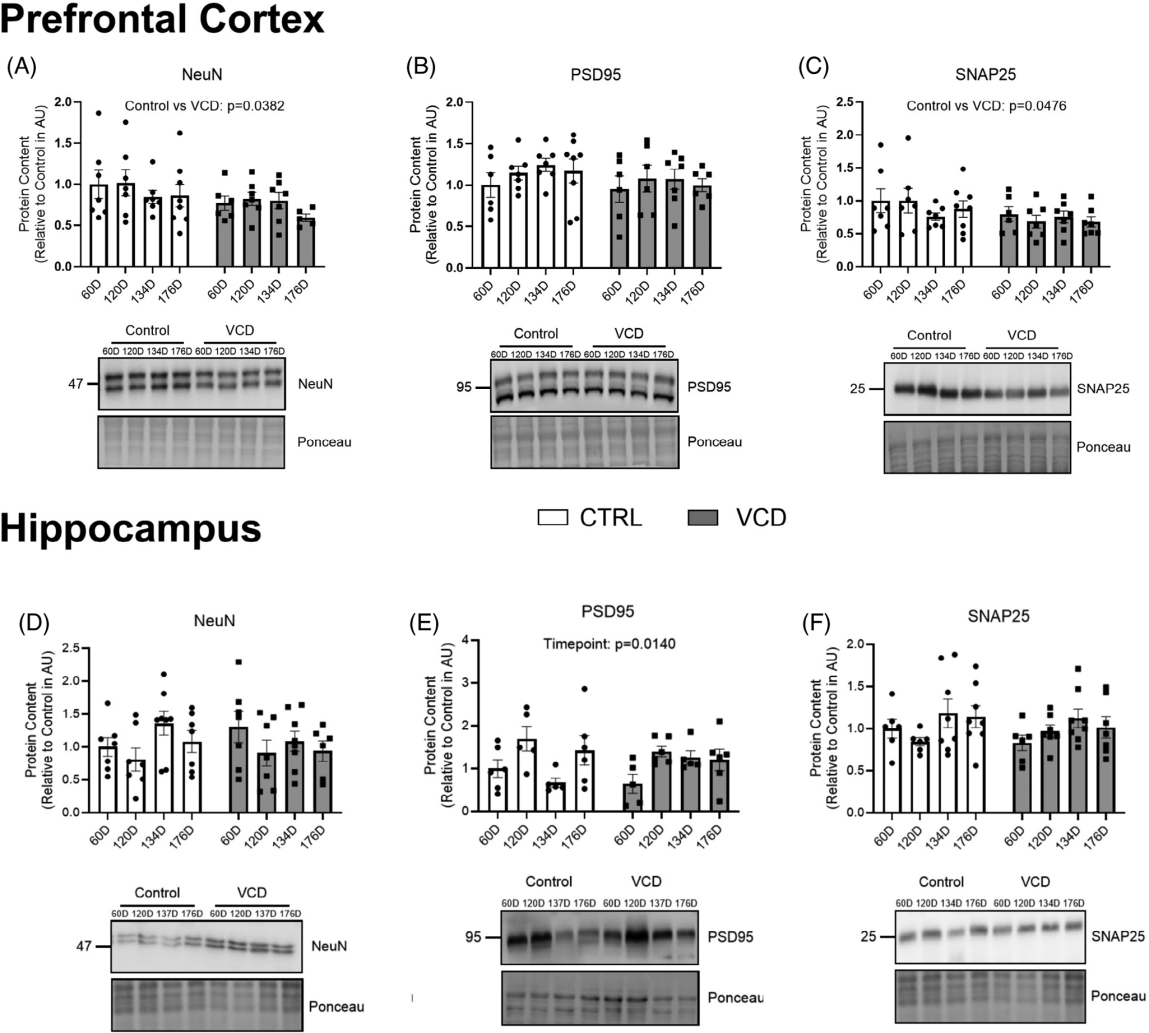
Content of PFC and HIPP neuronal and synaptic markers characterization of the model at different timepoints of VCD progression compared to control. A, NeuN content (AU). B, PSD95 content (AU). C, SNAP25 content (AU). D, NeuN content (AU). E, PSD95 content (AU). F, SNAP25 content (AU). All western blots are accompanied by representative blot images. All data were analyzed using a two‐way ANOVA. *n* = 7 per condition for western blots, *n* = 5 per condition for activity assays. ANOVA, analysis of variance; AU, arbitrary scale; BACE1, β‐secretase; CTRL, control; HIPP, hippocampus; PFC, prefrontal cortex; RFU, relative fluorescence unit; VCD, 4‐vinylcyclohexene diepoxide; 60D, 60 days post completion of injection; 120D, 120 days post completion of injection; 134D, 134 days post completion of injection; 176D, 176 days post completion of injection

Markers of mature neuron content (NeuN) and pre‐ (SNAP25) and post‐synaptic (PSD95) proteins were examine to investigate the effects of VCD on neuronal and synaptic outcomes. In the PFC there was a main effect of treatment where VCD mice had lower NeuN protein content regardless of timepoint (*P* = 0.0382; Figure 4A). PSD95 protein content was not different between groups (*P* > 0.05), while SNAP25 protein content was also lower in those injected with VCD (*P* = 0.0476, main effect; Figure 4B,C). In the HIPP, NeuN protein content was not different between groups (*P* > 0.05; Figure 4D). PSD95 content had a main effect of timepoint (*P* = 0.0140), where the 60D timepoint was different from the 120D timepoint (60D, *P* = 0.0201). There were no differences observed for SNAP25 protein content between all groups (*P* > 0.05; Figure 4E,F).

### 3.4 Effects of HIIT initiated at the onset of menopause (day 120 post‐VCD injection)

**FIGURE 5:**
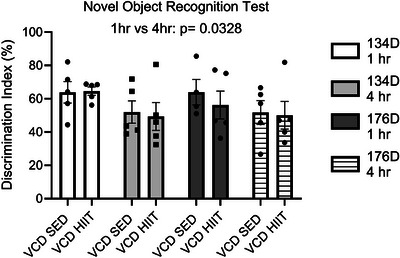
Exercise effects on cognitive testing. Novel object recognition testing results 134, and 176 days post‐injection using a 1 and 4 hour time interval (discrimination index %). All data were analyzed using a three‐way ANOVA. *n* = 5 per condition. ANOVA, analysis of variance; CTRL, control; VCD, 4‐vinylcyclohexene diepoxide; 134D, 134 days post completion of injection; 176D, 176 days post completion of injection

After 2 and 8 weeks of HIIT, recognition memory was evaluated via NORT. We found that 134 and 176 days post‐injection there were no differences in discrimination index % regardless of exercise status (*P* > 0.05; Figure 5). Comparing both timepoints at each test there was a main effect of inter‐trial interval time (*P* = 0.0328; Figure 5). These results demonstrate that when initiated at the onset of menopause, 8 weeks of HIIT does not improve recognition memory when measured by NORT in VCD‐injected mice.

### 3.5 APP processing and signalling

**FIGURE 6:**
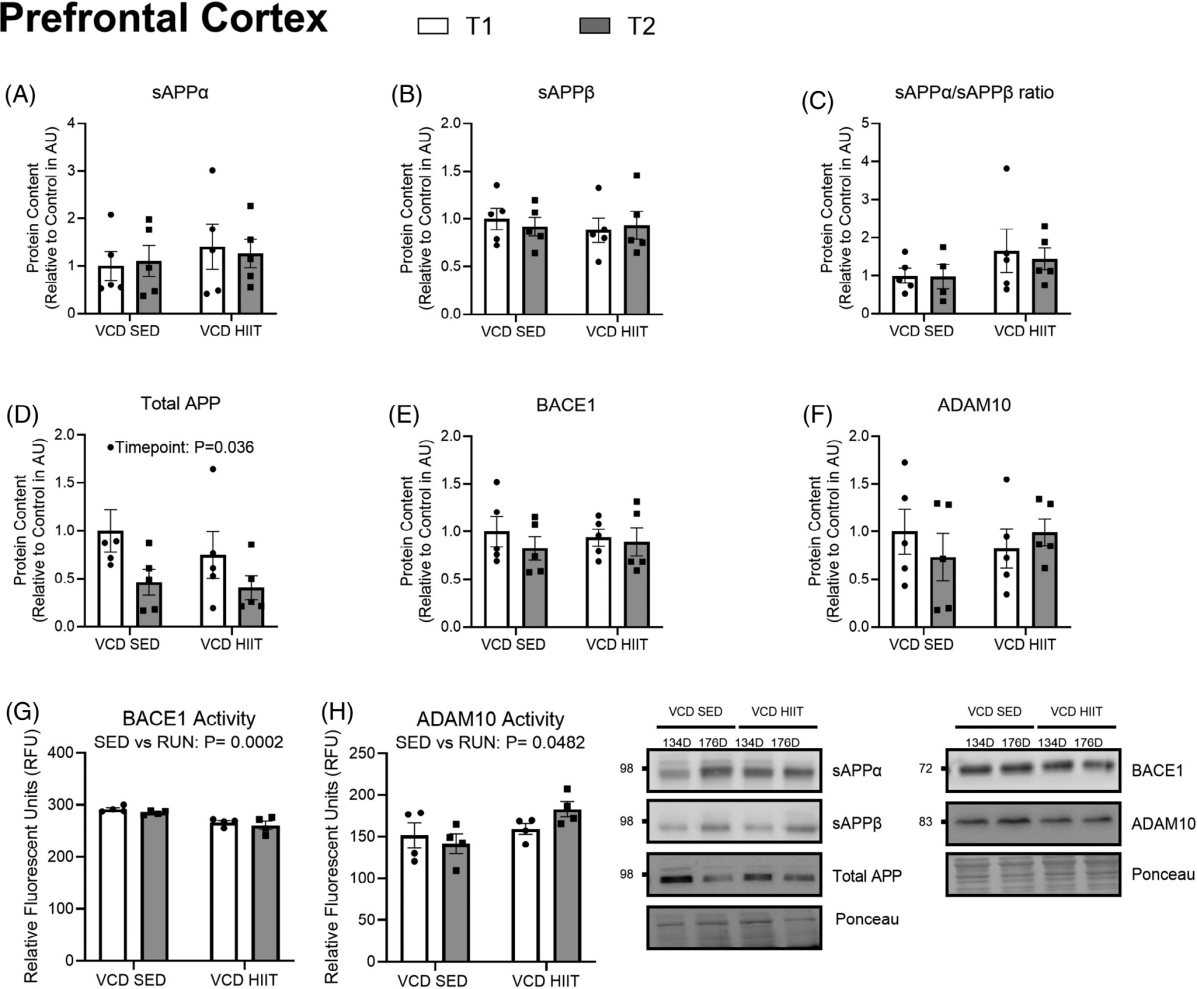
Content of PFC APP markers showing the effects of exercise in the model. A, sAPPα content (AU). B, sAPPβ content (AU). C, sAPPα/sAPPβ content ratio (AU). D, total APP content (AU). E, BACE1 content (AU). F, ADAM10 content (AU). G, BACE1 activity (RFU). H, ADAM10 activity (RFU). All blots are accompanied by representative blots. All data analyzed using a two‐way ANOVA. *n* = 5 per condition. ADAM10, a disintegrin and metalloprotease domain 10; ANOVA, analysis of variance; APP, amyloid precursor protein; AU, arbitrary scale; BACE1, β‐secretase; CTRL, control; HIPP, hippocampus; PFC, prefrontal cortex; RFU, relative fluorescence unit; VCD, 4‐vinylcyclohexene diepoxide; 134D, 134 days post completion of injection; 176D, 176 days post completion of injection

In the PFC, there was a main effect of timepoint for APP content, where there was a lower amount of total APP at the 176D timepoint regardless of exercise intervention (134D VCD sedentary [SED] 1.0 ± 0.5, VCD HIIT 0.75 ± 0.5, 176D VCD SED 0.46 ± 0.3, VCD HIIT 0.40 ± 0.3; *P* = 0.036; Figure 6D). There were no differences present between groups when looking at content of sAPPα, sAPPβ, BACE1, ADAM10, or sAPPα/sAPPβ content ratio (*P* > 0.05; Figures 6A–C,E,F). Enzymatic activity was also measured where it was shown that there was a main effect of exercise on BACE1 enzyme activity where it was lower in the groups who had exercised (134D VCD SED 291.6 ± 5.9, VCD HIIT 265.9 ± 7.4, 176D VCD SED 285.1 ± 4.0, VCD HIIT 260.7 ± 16.2; *P* = 0.0002; Figure 6G). There was a main effect of exercise on ADAM10 enzyme activity where it was higher in the exercise groups (134D VCD SED 151.6 ± 30.5, VCD HIIT 159.3 ± 13.4, 176D VCD SED 141.5 ± 23.5, VCD HIIT 182.9 ± 18.5; *P* = 0.0482; Figure 6H). These data show that HIIT can alter BACE1 and ADAM10 enzyme activity in the PFC after menopause.

**FIGURE 7:**
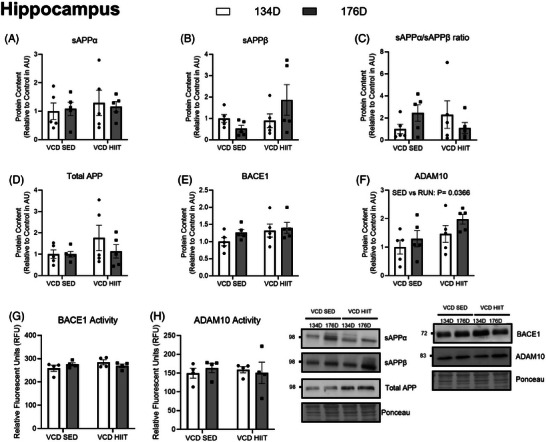
Content of HIPP APP markers showing the effects of exercise in the VCD model. A, sAPPα content (AU). B, sAPPβ content (AU). C, sAPPα/sAPPβ content ratio (AU). D, Total APP content (AU). E, BACE1 content (AU). F, ADAM10 content (AU). G, BACE1 activity (RFU). H, ADAM10 activity (RFU). All graphs are accompanied by representative blots. All data were analyzed using a two‐way ANOVA. *n* = 5 per condition. ADAM10, a disintegrin and metalloprotease domain 10; ANOVA, analysis of variance; APP, amyloid precursor protein; AU, arbitrary scale; BACE1, β‐secretase; CTRL, control; HIIT, high‐intensity interval training; HIPP, hippocampus; PFC, prefrontal cortex; RFU, relative fluorescence unit; SED, sedentary; T1, 134 days post completion of injection; T2, 176 days post completion of injection; VCD, 4‐vinylcyclohexene diepoxide

In the HIPP, protein content was not significantly different between groups when looking at total APP, sAPPα, sAPPβ, BACE1, the sAPPα/sAPPβ ratio, nor was BACE1 and ADAM10 enzyme activity affected by ovarian failure (*P* > 0.05; Figure 7A–E,G,I). However, there was a main effect of ADAM10 protein content where it was higher in the exercised VCD mice compared to the sedentary mice (134D VCD SED 1.0 ± 0.5, VCD HIIT 1.5 ± 0.07, 176D VCD SED 1.3 ± 0.6, VCD HIIT 2.0 ± 0.4; *P* = 0.0366; Figure 7F).

### 3.6 Neuronal and synaptic markers

**FIGURE 8:**
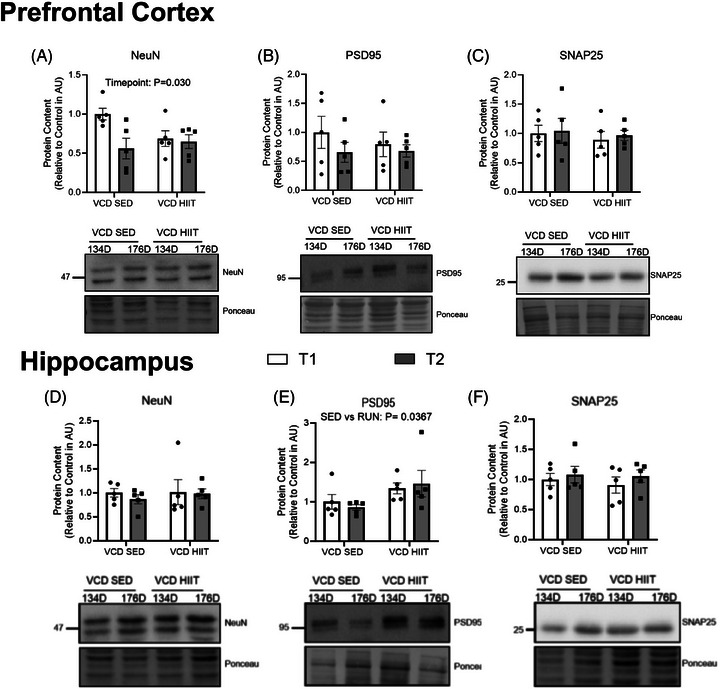
Content of PFC and HIPP neuronal and synaptic markers characterization of the model at different timepoints of VCD progression compared to control. A, NeuN content (AU). B, PSD95 content (AU). C, SNAP25 content (AU). D, NeuN content (AU). E, PSD95 content (AU). F, SNAP25 content (AU). All western blots are accompanied by representative blot images. All data were analyzed using a two‐way ANOVA. *n* = 7 per condition for western blots, *n* = 5 per condition for activity assays. ANOVA, analysis of variance; AU, arbitrary scale; CTRL, control; HIIT, high‐intensity interval training; HIPP, hippocampus; PFC, prefrontal cortex; SED, sedentary; VCD, 4‐vinylcyclohexene diepoxide; 60D, 60 days post completion of injection; 120D, 120 days post completion of injection; 134D, 134 days post completion of injection; 176D, 176 days post completion of injection

In the PFC, there was a main effect of time for NeuN protein content, where NeuN content was lower at the 176‐day timepoint compared to 134 days (*P* = 0.030; Figure 8A). PSD95 and SNAP25 protein content were not different between groups (*P* > 0.05; Figure 8B,C). In the HIPP, NeuN protein content was not different between groups (*P* > 0.05; Figure 8D). There was a main effect of exercise on PSD95 protein content where it was higher in the HIIT groups (Figure 8E). SNAP25 protein content, was not different between groups (*P* > 0.05; Figure 8F).

## 4 DISCUSSION

To develop preventative measures for menopause‐influenced AD, it is critical to use animal models that accurately replicate the physiology of average human menopause. The purpose of the current study was to investigate the effects of chemically induced ovarian failure in mice on time‐course adaptations in cognition, APP processing, and synaptic markers. The VCD‐treated mouse model, which induces gradual ovarian degeneration and estrogen depletion,^18^ mirrors human menopause and is a valuable model that allows for investigation of both the perimenopausal and menopausal periods. We further aimed to assess the effects of HIIT on cognition, APP processing, and synaptic markers when initiated at the onset of menopause. VCD resulted in lower recognition memory as assessed by the discrimination index in the NORT, starting in the perimenopausal phase and persisting post‐menopause. The VCD mice also had lower PFC NeuN and SNAP25 protein content alongside lower ADAM10 and higher BACE1 enzyme activity in the HIPP, all prior to the onset of menopause. These findings indicate that neuronal deficits of menopause are established throughout the perimenopausal transition. HIIT introduced during menopause did not alter recognition memory assessed via NORT or recover NeuN content; however, it did result in less BACE1 and more ADAM10 enzyme activity in the PFC. Furthermore, in the HIPP, HIIT intervention resulted in higher PSD95 and ADAM10 protein expression. These findings contribute to a better understanding of the neuronal importance of sex hormones, which ultimately facilitates our understanding of the cognitive impacts that menopause has on females. We further demonstrate the potential of HIIT to alter neuronal markers related to AD when initiated at menopause.

Similar to what is observed in OVX models,^4^, ^33^ VCD mice had lower active memory recall as measured through NORT compared to the control mice. The VCD model can provide further insight as this effect was seen in all timepoints, suggesting that the decline in cognition begins before ovarian failure is complete. Human literature has demonstrated that many females have cognitive impairments beginning during the perimenopausal transition and that continue to be present after the transition, which aligns with our findings.^12^, ^36^ The mechanisms that can be used to explain the lower cognition in the VCD model are unknown; however, our work aimed to examine some potential mechanisms related to synaptic proteins and APP processing.

NeuN and SNAP25 protein content were lower in the PFC of VCD mice and may provide a mechanistic basis for the observed cognitive effects. This lower content was found across all timepoints measured, implying that the factors were related to menopause. These changes may be demonstrative of altered mature neuron content (NeuN) and vesicle release (SNAP25) from neurons, which could affect cognition. Our previous work has demonstrated that OVX results in a shift in APP processing toward the amyloidogenic cascade.^4^, ^35^ Here we demonstrate that the VCD model impacted the activity of the competing APP enzymes BACE1 and ADAM10. In the HIPP, VCD mice had higher BACE1 activity at both the 60‐ and 176‐day timepoints, and lower ADAM10 activity at the 176‐day timepoint compared to the 60‐day timepoint. The higher enzymatic activity of BACE1 was the same across timepoints in the VCD‐treated groups, alongside lower levels of ADAM10, which could indicate a preferential degradation through the amyloidogenic cascade.

Imbalances between BACE1 and ADAM10 protein expression and enzyme activity where BACE1 is higher than ADAM10 may result in increased production and accumulation of Aβ peptides which can progress to cognitive decline and neurodegeneration. The VCD group had higher BACE1 activity (a marker of amyloidogenic production) and a lower ADAM10 activity (a competitive enzyme countering BACE1) in the HIPP. The higher BACE1 enzyme activity was present at both the 60‐ and 176‐day post‐injection timepoints in VCD mice. The ADAM10 activity was lower at the 176‐day timepoint in the VCD‐injected mice compared to the 60‐day timepoint showing that this effect is present only with complete follicle depletion. These findings indicate a potential time course of pathological change, when BACE1 changes are seen before alterations in ADAM10. Changes in BACE1 and ADAM10 may occur before significant Aβ accumulation is detectable, potentially allowing for the implementation of an early intervention. Our results provide support for targeting BACE1 early in life or in perimenopause to help mitigate Aβ accumulation.

In summary, the VCD model allows for the assessment of impacts from the gradual reductions in ovarian function that closely mimics the human menopausal experience.^17^, ^18^, ^19^ We demonstrate that cognitive and biochemical effects related to AD pathology are present from the earliest available timepoint, 60 days post‐VCD injections. This illustrates that the negative neuronal effects caused by VCD begin prior to what the model classifies as the “early perimenopausal” stage of progression. Future work may want to examine the effect of exercise at earlier timepoints. While the VCD model of menopause provides the opportunity to examine the progression through perimenopause and into menopause, there remain some limitations in that the use of a chemical to induce menopause may not perfectly replicate all aspects of natural menopause. While it is hypothesized that the reductions in memory and changes to protein markers in this study are due to the reduction in estrogen, it is likely that a combination of changes, including a relative imbalance in other hormones, are contributing to these outcomes. To fully elucidate the effects of reduced estrogens in the VCD model future work may want to include an estrogen‐treated group.

Exercise is known to improve memory and reduce the risk of AD. We investigated if 8 weeks of HIIT can mitigate the menopause‐associated changes in AD‐related markers. Our results show that HIIT can lower BACE1 and increase ADAM10 enzyme activity in the PFC. This is similar to what has been observed in OVX models with other exercise interventions,^5^, ^35^, ^37^ suggesting that exercise has a potential effect to reduce amyloidogenic processing of APP. PSD95 protein content was also higher in the HIPP of the HIIT mice, a synaptic marker associated with improved structural synaptic support.^38^ The higher PSD95 protein content could indicate a strengthened synapse as this scaffolding is essential to maintaining synaptic integrity.^38^, ^39^ These findings show that the neurological changes of VCD‐induced menopause can be at least partially reduced by exercise. HIIT was, however, not effective at recovering NeuN protein content in the PFC, as NeuN protein content was lower in the 176‐day timepoint in both VCD groups regardless of exercise status.

Previous work has demonstrated a positive effect of exercise on APP processing in which the cleavage of APP is pushed to the non‐amyloidogenic cascade.^24^, ^28^, ^35^, ^37^ In the current study, HIIT influenced BACE1 and ADAM10 regardless of timepoint of menopause. HIIT resulted in lower BACE1 enzyme activity and higher ADAM10 enzyme activity in the PFC. This could indicate a shifting of APP processing toward the non‐amyloidogenic cascade. When paired with a higher content of the ADAM10 protein in the HIPP of the HIIT groups, this further strengthens the beneficial impact of exercise on AD markers. Previous literature has shown that OVX and AD models have increased rates of BACE1 enzyme activity matched with decreases in that of ADAM10^32^, ^35^, ^40^ and that exercise has been shown to reduce BACE1 and increase ADAM10 enzyme activity.^32^, ^34^, ^35^ Although the results demonstrate the effectiveness of HIIT in altering APP processing, we did not observe any improvements in recognition memory. This highlights important limitations to this study. The use of a single cognitive test to evaluate memory may not provide a comprehensive view of the potential for HIIT to improve memory. The lack of memory improvements with HIIT may be due to the exercise duration, frequency, or intensity being too low. It is further possible that the timing of when HIIT was introduced may be important. Starting the HIIT intervention earlier, during the perimenopausal transition, might have yielded different results.

In conclusion, this study provides critical information on the effects VCD‐induced ovarian hormone depletion on active memory recall, APP processing, and synaptic markers. (1) This study demonstrated that VCD‐treated mice had a lower discrimination index compared to control mice during the NORT, starting before the onset of menopause. (2) PFC NeuN and SNAP25 protein content were lower in VCD mice at all timepoints. (3) These protein changes were accompanied by higher HIPP BACE1 activity and lower ADAM10 activity. Further, we demonstrate that HIIT increased PSD95 and ADAM10 protein content while reducing BACE1 activity and increasing ADAM10 activity in the PFC. Together, this work highlights the importance of examining the time course in the progression to menopause and the use of VCD as a model to investigate changes in the brain. Specifically, we have shown that cognitive and neurological changes are formed during the perimenopausal phase, which may lay the foundation for the increased risk in neurological conditions such as AD that disproportionately impact post‐menopausal females. Finally, exercise provides a means to blunt the effects of VCD‐induced estrogen loss on the brain; however, further work is required to elucidate the most effective combination of intensity, duration, and time of implementation.

## AUTHOR CONTRIBUTIONS

Conception and design of study: W. Glen Pyle, Geoffrey A. Power, and Rebecca E. K. MacPherson. Acquisition of data: Ahmad Mohammad, Michael S. Finch, Ciara Barry, Shawn M. Beaudette, and Rebecca E. K. MacPherson. Analysis and interpretation: Ahmad Mohammad, Michael S. Finch, Newman Sze, and Rebecca E. K. MacPherson. Drafting of the article: Ahmad Mohammad, Michael S. Finch, Newman Sze, and Rebecca E. K. MacPherson. Revising the article: all authors have contributed to revising the article. All authors have read and approved the manuscript.

## CONFLICT OF INTEREST STATEMENT

The authors declare no conflicts of interest. Author disclosures are available in the supporting information.

## Supporting information



Supporting Information
